# Supplementary material for: Biomarker Changes in Response to a 12-Week Supplementation of an Oral Nutritional Supplement Enriched with Protein, Vitamin D and HMB in Malnourished Community Dwelling Older Adults with Sarcopenia
Source: Nutrients. 2022 Mar 11;14(6):1196. doi: 10.3390/nu14061196 (PMC8953113; doi:10.3390/nu14061196)
Supplement: Supplementary file 1 [file nutrients-14-01196-s001.zip › nutrients-1616931-supplementary.pdf]

Table S1. Listing of biomarkers analyzed to identify which markers are impacted by chronic nutrition supplementation.

6Ckine (pg·mL^{-1})
Adiponectin (μg·mL^{-1})
Agouti-Related Protein (AgRP) (pg·mL^{-1})
Alanine Transaminase (ALT) (U·L^{-1})
Alpha-1-Antichymotrypsin (AACT) (μg·mL^{-1})
Alpha-1-Antitrypsin (AAT) (mg·mL^{-1})
Alpha-1-Microglobulin (A1Micro) (μg·mL^{-1})
Alpha-2-Macroglobulin (A2Macro) (mg·mL^{-1})
Alpha-Fetoprotein (AFP) (ng·mL^{-1})
Amphiregulin (AR) (pg·mL^{-1})
Angiopoietin-2 (ANG-2) (ng·mL^{-1})
Angiotensin Converting Enzyme (ACE) (ng·mL^{-1})
Angiotensinogen (ng·mL^{-1})
Apolipoprotein (a) (LP(a)) (μg·mL^{-1})
Apolipoprotein A-I (Apo A-1) (mg·mL^{-1})
Apolipoprotein A II (Apo A-II) (ng·mL^{-1})
Apolipoprotein A IV (Apo A-IV) (μg·mL^{-1})
Apolipoprotein B (Apo B) (μg·mL^{-1})
Apolipoprotein C I (Apo C-I) (ng·mL^{-1})
Apolipoprotein C III (Apo C-III) (μg·mL^{-1})
Apolipoprotein D (Apo D) (μg·mL^{-1})
Apolipoprotein E (Apo E) (μg·mL^{-1})
Apolipoprotein H (Apo H) (μg·mL^{-1})
AXL Receptor Tyrosine Kinase (AXL) (ng·mL^{-1})
Aspartate Aminotransferase (AST) (U·L^{-1})
B-cell Activating Factor (BAFF) (pg·mL^{-1})
B Lymphocyte Chemoattractant (BLC) (pg·mL^{-1})
Beta-2 Microglobulin (B2M) (μg·mL^{-1})
Betacellulin (BTC) (pg·mL^{-1})
Brain-Derived Neurotrophic Factor (BDNF) (ng·mL^{-1})
Blood Urea Nitrogen (BUN) (mg·dL^{-1})
C-Peptide (ng·mL^{-1})
C-Reactive Protein (CRP) (μg·mL^{-1})
Calbindin (ng·mL^{-1})
Cancer Antigen 125 (CA-125) (U·mL^{-1})
Cancer Antigen 15-3 (CA-15-3) (U·mL^{-1})
Cancer Antigen 19-9 (CA-19-9) (U·mL^{-1})
Carcinoembryonic Antigen (CEA) (ng·mL^{-1})
CD40 Ligand (CD40-L) (ng·mL^{-1})
CD5 Antigen-like (CD5L) (ng·mL^{-1})
Chemokine CC-4 (HCC-4) (ng·mL^{-1})
Chloride (mmol·L^{-1})
Chromogranin-A (CgA) (ng·mL^{-1})
Ciliary Neurotrophic Factor (CNTF) (pg·mL^{-1})
Clusterin (CLU) (μg·mL^{-1})
Complement C3 (C3) (μg·mL^{-1})
Complement Factor H-Related Protein (CFHR1) (μg·mL^{-1})
Cortisol (ng·mL^{-1})

Creatine Kinase-MB (CK-MB) ($ng \cdot mL^{-1}$)
Cystatin-C ($ng \cdot mL^{-1}$)
Dehydroepiandrosterone-sulphate (DHEA-S) ($\mu g \cdot dL^{-1}$)
E-Selectin ($ng \cdot mL^{-1}$)
EN-RAGE ($\mu g \cdot mL^{-1}$)
Eotaxin-1 ($pg \cdot mL^{-1}$)
Eotaxin-2 ($pg \cdot mL^{-1}$)
Eoxtaxin-3 ($pg \cdot mL^{-1}$)
Epidermal Growth Factor (EGF) ($pg \cdot mL^{-1}$)
Epidermal Growth Factor Receptor (EGFR) ($ng \cdot mL^{-1}$)
Epiregulin (EPR) ($pg \cdot mL^{-1}$)
Epithelial-Derived Neutrophil-Activating Protein 78 (ENA-78) ($ng \cdot mL^{-1}$)
Estradiol ($pg \cdot ml^{-1}$)
Factor VII ($ng \cdot mL^{-1}$)
Fas Ligand (FasL) ($pg \cdot mL^{-1}$)
FASLG Receptor ($ng \cdot mL^{-1}$)
Fatty Acid-Binding Protein, heart (F ABP, heart) ($ng \cdot mL^{-1}$)
Ferritin (FRTN) ($ng \cdot mL^{-1}$)
Fetuin-A ($\mu g \cdot mL^{-1}$)
Fibrinogen ($mg \cdot mL^{-1}$)
Fibroblast Growth Factor 4 (FGF-4) ($pg \cdot mL^{-1}$)
Fibroblast Growth Factor basic (FGF-basic) ($\mu g \cdot mL^{-1}$)
Follicle Stimulating Hormone (FSH) ($mIU \cdot mL^{-1}$)
Glucagon ($pg \cdot mL^{-1}$)
Glucagon-like Peptide-1 (GLP-1) ($pg \cdot mL^{-1}$)
Glutathione S-Transferase alpha (GST-alpha) ($ng \cdot mL^{-1}$)
Granulocyte Colony-Stimulating Factor (G-CSF) ($pg \cdot mL^{-1}$)
Granulocyte-Macrophage Colony-Stimulating Factor (GM-CSF) ($pg \cdot mL^{-1}$)
Growth Hormone (GH) ($ng \cdot mL^{-1}$)
Growth-Regulated Alpha Protein (GRO-alpha) ($pg \cdot mL^{-1}$)
Haptoglobin ($mg \cdot mL^{-1}$)
Heat Shock Protein 60 (HSP-60) ($ng \cdot mL^{-1}$)
Heparin-Binding EGF-Like Growth Factor (HB-EGF) ($pg \cdot mL^{-1}$)
Hepatocyte Growth Factor (HGF) ($ng \cdot mL^{-1}$)
Human Chorionic Gonadotropin beta (hCG) ($mIU \cdot mL^{-1}$)
Immunoglobulin A (IgA) ($mg \cdot mL^{-1}$)
Immunoglobulin E (IgE) ($U \cdot mL^{-1}$)
Immunoglobulin M (IgM) ($mg \cdot mL^{-1}$)
Insulin ($uIU \cdot mL^{-1}$)
Insulin-like Growth Factor 1 (IGF-1) ($ng \cdot mL^{-1}$)
Insulin-like Growth Factor Binding Protein 2 (IGFBP-2) ($ng \cdot mL^{-1}$)
Intercellular Adhesion Molecule 1 (ICAM-1) ($ng \cdot mL^{-1}$)
Interferon gamma (IFN-gamma) ($pg \cdot mL^{-1}$)
Interferon gamma Induced Protein 10 (IP10) ($pg \cdot mL^{-1}$)
Interferon-inducible T-cell alpha chemoattractant (ITAC) ($pg \cdot mL^{-1}$)
Interleukin-1 alpha (IL-1 alpha) ($ng \cdot mL^{-1}$)
Interleukin-1 beta (IL-1 beta) ($pg \cdot mL^{-1}$)
Interluekin-1 Receptor Antagonist (IL-1ra) ($pg \cdot mL^{-1}$)
Interleukin-2 (IL-2) ($pg \cdot mL^{-1}$)
Interleukin-2 Receptor alpha (IL-2ra) ($pg \cdot mL^{-1}$)

Interluekin-3 (IL-3) (ng·mL^{-1})
Interluekin-4 (IL-4) (pg·mL^{-1})
Interluekin-5 (IL-5) (pg·mL^{-1})
Interluekin-6 (IL-6) (pg·mL^{-1})
Interleukin-6 Receptor (IL-6r) (ng·mL^{-1})
Interluekin-7 (IL-7) (pg·mL^{-1})
Interleukin-8 (IL-8) (pg·mL^{-1})
Interluekin-10 (IL-10) (pg·mL^{-1})
Interleukin-12 Subunit p40 (IL-12p40) (ng·mL^{-1})
Interleukin-12 Subunit p70 (IL-12p70) (pg·mL^{-1})
Interluekin-13 (IL-13) (pg·mL^{-1})
Interluekin-15 (IL-15) (ng·mL^{-1})
Interleukin-16 (IL-16) (pg·mL^{-1})
Interluekin-17 (IL-17) (pg·mL^{-1})
Interleukin-18 (IL-18) (pg·mL^{-1})
Interleukin-23 (IL-23) (ng·mL^{-1})
Kidney Injury Molecule-1 (KIM-1) (ng·mL^{-1})
Latency-Associated Peptide of Transforming Growth Factor beta 1 (LAP TGF-b1) (ng·mL^{-1})
Lectin-like Oxidized LDL Receptor 1 (LOX-1) (ng·mL^{-1})
Leptin (ng·mL^{-1})
Luteinizing Hormone (LH) (mIU·mL^{-1})
Macrophage Colony-Stimulating Factor 1 (M-CSF) (ng·mL^{-1})
Macrophage-Derived Chemokine (MDC) (pg·mL^{-1})
Macrophage Inflammatory Protein-1 alpha (MIP-1 alpha) (pg·mL^{-1})
Macrophage Inflammatory Protein-1 beta (MIP-1 beta) (pg·mL^{-1})
Macrophage Inflammatory Protein-3 alpha (MIP-3 alpha) (pg·mL^{-1})
Macrophage Inflammatory Protein-3 beta (MIP-3 beta) (pg·mL^{-1})
Macrophage Migration Inhibitory Factor (MIF) (ng·mL^{-1})
Magnesium (mEq·L^{-1})
Malondialdehyde-Modified Low-Density Lipoprotein (MDA-LDL) (ng·mL^{-1})
Matrix Metalloproteinase-1 (MMP-1) (ng·mL^{-1})
Matrix Metalloproteinase-3 (MMP-3) (ng·mL^{-1})
Matrix Metalloproteinase-7 (MMP-7) (ng·mL^{-1})
Matrix Metalloproteinase-9 (MMP-9) (ng·mL^{-1})
Matrix Metalloproteinase-9, total (MMP-9 total) (ng·mL^{-1})
Matrix Metalloproteinase-10 (MMP-10) (ng·mL^{-1})
MHC Class I Chain-related protein A (MICA) (pg·mL^{-1})
Monocyte Chemotactic Protein-1 (MCP-1) (pg·mL^{-1})
Monocyte Chemotactic Protein-2 (MCP-2) (pg·mL^{-1})
Monocyte Chemotactic Protein-3 (MCP-3) (pg·mL^{-1})
Monocyte Chemotactic Protein-4 (MCP-4) (pg·mL^{-1})
Monokine Induced by Gamma Interferon (MIG) (pg·mL^{-1})
Myeloid Progenitor Inhibitory Factor 1 (MPIF-1) (ng·mL^{-1})
Myeloperoxidase (MPO) (ng·mL^{-1})
Myoglobin (ng·mL^{-1})
N-terminal Prohormone of Brain Natriuretic Peptide (NT proBNP) (pg·mL^{-1})
Nerve Growth Factor beta (NGF-beta) (ng·mL^{-1})
Neuron Specific Enolase (NSE) (ng·mL^{-1})
Neuronal Cell Adhesion Molecule (Nr-CAM) (ng·mL^{-1})
Neutrophil Gelatinase-Associated Lipocalin (NGAL) (ng·mL^{-1})

Osteopontin (ng·mL^{-1})
Osteoprotegerin (OPG) (pM)
Pancreatic Polypeptide (PPP) (pg·mL^{-1})
Peptide YY (PYY) (pg·mL^{-1})
Phosphate (mg·dL^{-1})
Placenta Growth Factor (PLGF) (pg·mL^{-1})
Plasminogen Activator Inhibitor 1 (PAI-1) (ng·mL^{-1})
Platelet-Derived Growth Factor BB (PDGF-BB) (pg·mL^{-1})
Pre-albumin (mg·dL^{-1})
Progesterone (ng·mL^{-1})
Proinsulin, Intact (pM)
Proinsulin, Total (pM)
Prolactin (PRL) (ng·mL^{-1})
Prostate-Specific Antigen, Free (PSA-f) (ng·mL^{-1})
Pulmonary and Activation-Regulated Chemokine (PARC) (ng·mL^{-1})
Receptor for Advanced Glycosylation End Products (RAGE) (ng·mL^{-1})
Red Blood Cells (RBC) (x10^{12}·L^{-1})
Resistin (ng·mL^{-1})
S100 Calcium-Binding Protein B (S100-B) (ng·mL^{-1})
Serotransferrin (Transferrin) (mg·dL^{-1})
Serum Amyloid P-Component (SAP) (μg·mL^{-1})
Sex Hormone Binding Globulin (SHBG) (mol·L^{-1})
Sortilin (ng·mL^{-1})
Stem Cell Factor (SCF) (pg·mL^{-1})
Stromal Cell Derived Factor-1 (SDF-1) (pg·mL^{-1})
Superoxide Dismutase-1 (SOD-1) (ng·mL^{-1})
Total c-Terminal Agrin Fragment (t-CAF) (pmols)
T-cell-Specific Protein RANTES (RANTES) (ng·mL^{-1})
T Lymphocyte-Secreted Protein I-309 (I-309) (pg·mL^{-1})
Tamm Horsfall Urinary Glycoprotein (THP) (μg·mL^{-1})
Tenascin-C (TN-C) (ng·mL^{-1})
Testosterone (ng·mL^{-1})
Thrombomodulin (TM) (ng·mL^{-1})
Thrombospondin-1 (ng·mL^{-1})
Thyroid Stimulating Hormone (TSH) (μIU·mL^{-1})
Thyroxine Binding Globulin (TBG) (μg·mL^{-1})
Tissue Inhibitor of Metalloproteinases 1 (TIMP-1) (ng·mL^{-1})
TNF-Related Apoptosis-Inducing Ligand Receptor (TRAIL-R3) (ng·mL^{-1})
Total Protein (g·dL^{-1})
Transforming Growth Factor alpha (TGF alpha) (pg·mL^{-1})
Transforming Growth Factor beta-3 (TGF-beta-3) (pg·mL^{-1})
Transthyretin (TTR) (mg·mL^{-1})
Trefoil Factor-3 (TFF3) (μg·mL^{-1})
Triglycerides (mg·dL^{-1})
Tumor Necrosis Factor alpha (TNF-alpha) (pg·mL^{-1})
Tumor Necrosis Factor beta (TNF-beta) (pg·mL^{-1})
Tumor Necrosis Factor Receptor-1 (TNFR1) (pg·mL^{-1})
Tumor Necrosis Factor Receptor-2 (TNFR2) (ng·mL^{-1})
Vascular Cell Adhesion Molecule (VCAM-1) (ng·mL^{-1})
Vascular Endothelial Growth Factor (VEGF) (pg·mL^{-1})

Vitamin B12 (pg·mL^{-1})
Vitamin D (nmol·L^{-1})
Vitamin D-Binding Protein (VDBP) (μg·mL^{-1})
Vitamin E (nmol·mL^{-1})
Vitamin K-Dependent Protein S (VKDPS) (μg·mL^{-1})
Vitronectin (μg·mL^{-1})
Von Willebrand Factor (VSF) (μg·mL^{-1})

Table S2. Biomarkers excluded from statistical analyses because at least 30% of the samples had levels below the lower limit of quantitation.

Agouti-Related Protein (AgRP) (pg·mL^{-1})
Amphiregulin (AR) (pg·mL^{-1})
B Lymphocyte Chemoattractant (BLC) (pg·mL^{-1})
Betacellulin (BTC) (pg·mL^{-1})
Calbindin (ng·mL^{-1})
Cancer Antigen 125 (CA-125) (U·mL^{-1})
Cancer Antigen 19-9 (CA-19-9) (U·mL^{-1})
Ciliary Neurotrophic Factor (CNTF) (pg·mL^{-1})
Eotaxin-1 (pg·mL^{-1})
Eoxtaxin-3 (pg·mL^{-1})
Epiregulin (EPR) (pg·mL^{-1})
Fas Ligand (FasL) (pg·mL^{-1})
Fatty Acid-Binding Protein, heart (F ABP, heart) (ng·mL^{-1})
Fibrinogen (mg·mL^{-1})
Fibroblast Growth Factor 4 (FGF-4) (pg·mL^{-1})
Fibroblast Growth Factor basic (FGF-basic) (μg·mL^{-1})
Glucagon (pg·mL^{-1})
Glucagon-like Peptide-1 (GLP-1) (pg·mL^{-1})
Glutathione S-Transferase alpha (GST-alpha) (ng·mL^{-1})
Granulocyte Colony-Stimulating Factor (G-CSF) (pg·mL^{-1})
Granulocyte-Macrophage Colony-Stimulating Factor (GM-CSF) (pg·mL^{-1})
Heat Shock Protein 60 (HSP-60) (ng·mL^{-1})
Heparin-Binding EGF-Like Growth Factor (HB-EGF) (pg·mL^{-1})
Human Chorionic Gonadotropin beta (hCG) (mIU·mL^{-1})
Immunoglobulin E (IgE) (U·mL^{-1})
Interferon gamma (IFN-gamma) (pg·mL^{-1})
Interleukin-1 beta (IL-1 beta) (pg·mL^{-1})
Interluekin-1 Receptor Antagonist (IL-1ra) (pg·mL^{-1})
Interleukin-2 (IL-2) (pg·mL^{-1})
Interluekin-3 (IL-3) (ng·mL^{-1})
Interluekin-4 (IL-4) (pg·mL^{-1})
Interluekin-5 (IL-5) (pg·mL^{-1})
Interluekin-6 (IL-6) (pg·mL^{-1})
Interluekin-7 (IL-7) (pg·mL^{-1})
Interluekin-10 (IL-10) (pg·mL^{-1})
Interleukin-12 Subunit p70 (IL-12p70) (pg·mL^{-1})

Interluekin-13 (IL-13) (pg·mL^{-1})
Interluekin-15 (IL-15) (ng·mL^{-1})
Interluekin-17 (IL-17) (pg·mL^{-1})
Lectin-like Oxidized LDL Receptor 1 (LOX-1) (ng·mL^{-1})
MHC Class I Chain-related protein A (MICA) (pg·mL^{-1})
Macrophage Inflammatory Protein-1 alpha (MIP-1 alpha) (pg·mL^{-1})
Macrophage Inflammatory Protein-1 beta (MIP-1 beta) (pg·mL^{-1})
Malondialdehyde-Modified Low-Density Lipoprotein (MDA-LDL) (ng·mL^{-1})
Matrix Metalloproteinase-9 (MMP-9) (ng·mL^{-1})
Monocyte Chemotactic Protein-3 (MCP-3) (pg·mL^{-1})
Nerve Growth Factor beta (NGF-beta) (ng·mL^{-1})
Peptide YY (PYY) (pg·mL^{-1})
Placenta Growth Factor (PLGF) (pg·mL^{-1})
Progesterone (ng·mL^{-1})
Proinsulin, Intact (pM)
Proinsulin, Total (pM)
Prostate-Specific Antigen, Free (PSA-f) (ng·mL^{-1})
S100 Calcium-Binding Protein B (S100-B) (ng·mL^{-1})
T Lymphocyte-Secreted Protein I-309 (I-309) (pg·mL^{-1})
Transforming Growth Factor alpha (TGF alpha) (pg·mL^{-1})
Transforming Growth Factor beta-3 (TGF-beta-3) (pg·mL^{-1})
Tumor Necrosis Factor alpha (TNF-alpha) (pg·mL^{-1})
Tumor Necrosis Factor beta (TNF-beta) (pg·mL^{-1})

Table S3. Means ± standard deviations (SD) of biomarkers that changed over 12 weeks after supplementation of the Experimental ONS (EONS) with HMB.

	EONS			CONS		
	Baseline	12 Weeks	Percent Change (n=90) [a]	Baseline	12 Weeks	Percent Change (n=103)
Osteopontin (ng·mL^{-1})	17.47 ± 15.84	14.67 ± 15.86	-18.24 ± 24.32 [c]	15.37 ± 8.63	14.04 ± 10.18	-4.59 ± 45.96
Ferritin (ng·mL^{-1})	127.42 ± 99.51	105.56 ± 105.18	-17.73 ± 26.36 [c]	136.95 ± 157.41	126.11 ± 162.06	-1.80 ± 48.70
Interleukin-6 Receptor (ng·mL^{-1})	25.89 ± 8.65	26.96 ± 9.20	4.29 ± 8.57 [c]	26.14 ± 8.08	26.75 ± 9.73	2.78 ± 9.04
Interluekin-16 (pg·mL^{-1})	383.56 ± 119.11	410.61 ± 137.97	9.16 ± 22.37 [b]	367.06 ± 132.91	374.79 ± 132.10	3.97 ± 16.99
Tumor Necrosis Factor receptor-1 (pg·mL^{-1})	2073.73 ± 857.29	2163.82 ± 896.57	5.04 ± 12.32 [b]	2064/73 ± 1090.94	2156.73 ± 1137.51	5.26 ± 15.52
Tumor Necrosis Factor receptor-2 (ng·mL^{-1})	7.44 ± 2.66	7.86 ± 3.08	6.48 ± 16.13 [b]	7.76 ± 2.98	8.09 ± 3.50	4.13 ± 16.99
Immunoglobulin A (mg·mL^{-1})	2.82 ± 1.53	2.98 ± 1.66	6.46 ± 12.21 [c]	2.51 ± 1.64	2.60 ± 1.78	3.53 ± 10.66
Immunoglobulin M (mg·mL^{-1})	1.45 ± 0.90	1.57 ± 0.92	11.40 ± 16.89 [c]	1.57 ± 1.32	1.62 ± 1.30	4.97 ± 14.94
Magnesium (mEq·L^{-1})	1.66 ± 0.18	1.71 ± 0.19	3.35 ± 6.31 [c]	1.70 ± 0.14	1.72 ± 0.15	1.02 ± 7.02
Total Protein (g·dL^{-1})	6.93 ± 0.41	7.05 ± 0.41	1.86 ± 4.56 [b]	6.98 ± 0.37	7.08 ± 0.40	1.52 ± 4.72
Vitamin E (nmol·mL^{-1})	15.86 ± 16.17	17.54 ± 18.14	15.15 ± 36.05 [b]	18.21 ± 23.16	22.03 ± 27.54	34.93 ± 110.28
Sex Hormone Binding Globulin (mol·L^{-1}) [a]	71.38 ± 39.56	60.84 ± 35.04	-11.59 ± 18.98 [c]	66.42 ± 33.92	58.57 ± 31.28	-9.95 ± 28.36
Myoglobin (ng·mL^{-1})	63.73 ± 50.62	71.78 ± 64.59	13.02 ± 30.80 [b]	53.57 ± 36.61	54.99 ± 33.54	8.25 ± 29.50

[a] EONS n=89 for Sex Hormone Binding Globulin
[b] Change from baseline using univariable dependent t-test with Sidak adjusted p-value ≤ 0.05, to account for the larger number of simultaneous tests.
[c] Change from baseline using univariable dependent t-test with Sidak adjusted p-value ≤ 0.001.

Table S4. Baseline biomarkers Correlation of biomarkers with measures of skeletal muscle strength at baseline in study participants (n=193).

	**Osteopontin**	**IGF-1**	**IGFBP-2**	**Leptin**	**SHBG**
Handgrip Strength (kg)	-0.169*	0.387*	-0.129	-0.221*	-0.271*
Average Extensor Peak Torque (Nm)	-0.156*	0.362*	-0.178*	-0.213*	-0.265*
Peak Extensor Peak Torque (Nm)	-0.157*	0.365*	-0.182*	-0.222*	-0.269*

* Indicates a significant correlation, $p < 0.05$.
Insulin Growth Factor-1 (IGF-1), Insulin Growth Factor Binding Protein-2 (IGFBP-2), Sex Hormone Binding Globulin (SHBG).
